# Influenza A (H3N2) Outbreak, Nepal

**DOI:** 10.3201/eid1108.050302

**Published:** 2005-08

**Authors:** Luke T. Daum, Michael W. Shaw, Alexander I. Klimov, Linda C. Canas, Elizabeth A. Macias, Debra Niemeyer, James P. Chambers, Robert Renthal, Sanjaya K. Shrestha, Ramesh P. Acharya, Shankar P. Huzdar, Nirmal Rimal, Khin S. Myint, Philip Gould

**Affiliations:** * Air Force Institute for Operational Health, Brooks City Base, San Antonio, Texas;; † University of Texas, San Antonio, Texas;; ‡ Centers for Disease Control and Prevention, Atlanta, Georgia;; § Walter Reed/Armed Forces Research Institute of Medical Sciences Research Unit-Nepal, Kathmandu, Nepal;; ¶ Association of Medical Doctors of Asia-Nepal, Kathmandu, Nepal;; # US Army Medical Component of the Armed Forces Research Institute of Medical Sciences, Bangkok, Thailand

**Keywords:** Influena A, H3N2, Hemagglutinin, genetic drift, variant

## Abstract

In July 2004, an outbreak of influenza A (H3N2) was detected at 3 Bhutanese refugee camps in southeastern Nepal. Hemagglutination inhibition showed that ≈40% of the viruses from this outbreak were antigenically distinct from the A/Wyoming/3/03 vaccine strain. Four amino acid differences were observed in most of the 26 isolates compared with the A/Wyoming/3/2003 vaccine strain. All 4 substitutions are located within or adjacent to known antibody-binding sites. Several isolates showed a lysine-to-asparagine substitution at position 145 (K145N) in the hemagglutinin molecule, which may be noteworthy since position 145 is located within a glycosylation site and adjacent to an antibody-binding site. H3N2 viruses continue to drift from the vaccine strain and may remain as the dominant strains during the 2005–2006 influenza season. Thus, the 2005–2006 Northern Hemisphere vaccine strain was changed to A/California/7/2004, a virus with all 4 amino acid substitutions observed in these Nepalese isolates.

## Abstract 2

Worldwide emergence of variant viruses has prompted a change in the 2005–2006 H3N2 influenza A vaccine strain.

The 2003–2004 influenza season was severe in terms of its impact on illness because of widespread circulation of antigenically distinct influenza A (H3N2) Fujian-like viruses. These viruses first appeared late during the 2002–2003 influenza season and continued to persist as the dominant circulating strain throughout the subsequent 2003–2004 influenza season, replacing the A/Panama/2007/99-like H3N2 viruses (1). Of the 172 H3N2 viruses genetically characterized by the Department of Defense in 2003–2004, only 1 isolate (from Thailand) belonged to the A/Panama-like lineage. In February 2003, the World Health Organization (WHO) changed the H3N2 component for the 2004–2005 influenza vaccine to afford protection against the widespread emergence of Fujian-like viruses (2). The annually updated trivalent vaccine consists of hemagglutinin (HA) surface glycoprotein components from influenza H3N2, H1N1, and B viruses.

The HA1 segment of the influenza HA protein is the most rapidly evolving gene product (3) and plays a major role in viral attachment and evasion from the adaptive immune response. Previous studies have demonstrated 5 antigenic sites on the HA1 polypeptide where antibody binding can occur (4,5). Additionally, several studies have documented specific immunodominant codons corresponding to specific amino acids of the HA protein that are directly involved in the divergence of antigenically distinct influenza viruses (6–8).

In July 2004, an outbreak of influenza A (H3N2) was detected in patients at 3 Bhutanese refugee camps in southeastern Nepal. To elucidate the molecular mechanism underlying the emergence of this H3N2 outbreak, we conducted a molecular analysis of the HA1 region of the HA protein. In this report, we describe the epidemiologic and molecular aspects of isolates obtained from this off-season influenza A (H3N2) outbreak.

## Materials and Methods

### Sample Collection and Antigenic Analysis

Sixty-four patients in Nepal that met US Department of Defense enrollment criteria (9) for influenzalike illness were evaluated by using onsite rapid influenza tests (Optical Immunoassay Rapid Diagnostic Tests, Thermo Electron Corp., San Jose, CA, USA) according to the manufacturer's instructions. Throat swab specimens were collected within the first 72 hours of onset of symptoms, routed through the Armed Forces Research Institute for Medical Sciences in Bangkok, Thailand, and shipped on dry ice to Brooks City Base in San Antonio, Texas, for clinical characterization and diagnosis using traditional culturing techniques and monoclonal antibody staining (10). Antigenic analysis of select isolates was performed at the Centers for Disease Control and Prevention (CDC) in Atlanta, Georgia, by using the hemagglutination inhibition (HI) assay and postinfection ferret antisera (11).

### Molecular Analysis

RNA was extracted from 48-hour shell vial cultures (10) by using the MagnaPure LX (Roche Molecular, Mannheim, Germany) and RNA Isolation Kit II (Roche Molecular) according to the manufacturer's protocols. For reverse transcription-polymerase chain reaction (RT-PCR) amplification, 5 μL RNA was added to a 50-μL master mixture containing 1× reaction buffer, 1.6 mmol/L MgSO_4_, 1× enzyme mixture, and 400 nmol/L primers (H3-F7, 5´-ACT-ATC-ATT-GCT-TTG-AGC-3´ and H3R-1184, 5´-ATG-GCT-GCT-TGA-GTG-CTT-3´) by using the SuperScript III One-Step RT-PCR System (Invitrogen, Carlsbad, CA, USA). PCR thermocycling consisted of an RT step at 50°C for 30 min, hot start activation at 95°C for 3 min, followed by 40 amplification cycles of 95°C for 30 s, 52°C for 15 s, and 68°C for 1 min, with a final extension cycle at 68°C for 7 min. All PCR products were visualized after electrophoresis in 2% precast gels stained with ethidium bromide (Invitrogen) under UV illumination. PCR products were purified by using the PCR Purification Kit (Qiagen Inc., Valencia, CA, USA). The HA1 amplicon (1177 bp) was sequenced by using the H3-F7 and H3R-1184 PCR primers (described above) and 2 additional internal oligonucleotides, H3R-466 (5´-GGT-GCA-ACC-AAT-TCA-ATC-3´) and H3F-282 (5´-CAG-CAA-CTG-TTA-CCC-3´).

Unincorporated fluorescent nucleotides were removed by using a Dye Ex 96-well plate kit (Qiagen) according to the manufacturer's recommendations. Nucleotide sequencing was performed by using the Big Dye Terminator v3.1 Kit and analyzed by using an ABI 3100 Genetic Analyzer (both from Applied Biosystems, Foster City, CA, USA) according to the manufacturer's specifications. Multiple sequence alignments, protein translation, and phylogenetic analysis were performed with the DNAStar (DNAStar Inc., Madison, WI, USA) software package. Three-dimensional HA protein structures were generated by using MOLMOL (12) and the Swiss-PDB Viewer programs (13). HA nucleotide sequences for all 26 Nepal isolates depicted in the phylogenetic analysis are available from GenBank under accession nos. AY945263–AY945288.

## Results

### Epidemiologic and Laboratory Assessment

**Figure 1:**
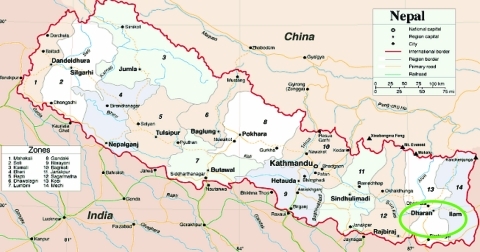
Early outbreak of influenza A (H3N2) in southeastern Nepal. The green circle shows the location of 3 Bhutan refugee camps where the outbreak occurred in early July 2004. (Map courtesy of http://www.maps.com)

Clinical evaluations and throat specimens were obtained from 64 patients from 3 refugee camps in southeastern Nepal (Figure 1). Of the 64 patients, 61 were refugees from Bhutan, 1 was a foreign aid worker from Japan, and 2 were Nepalese nationals. Most of the patients were <10 years of age; 36 were male and 28 were female. None had previously been vaccinated against influenza and of the 64 specimens collected, 42 (66%) tested positive for influenza A by culture.

### Antigenic Analysis

HI was performed by using postinfection ferret antisera with reference antigens that included the 2004–2005 H3N2 vaccine seed strain (A/Wyoming/03/2003) and the 2005–2006 Southern Hemisphere H3N2 vaccine strain (A/Wellington/1/2004). When compared with A/Wyoming/03/2003, 4 of 9 Nepal isolates showed 4-fold lower titers (1:320 versus 1:1,280 HI units) than that allowed for homologous titer of the reference antisera. This indicated that these 4 isolates were antigenically distinct. Six of 9 Nepal isolates were antigenically distinct when compared with the A/Wellington/1/2004 strain and showed a 4-fold (1:160 versus 1:640) reduction in titer to ferret antisera (Table 1).

**Table 1:** Hemagglutination inhibition (HI) reciprocal titers of influenza A (H3N2) viruses with ferret antisera*

Strain designation	Reference ferret antisera						
	PAN/2007	KO/770	WY/03	TX/40	OK/8	WEL/01	Date collected
Reference antigens							
A/Panama/2007/99	2,560	160	640	320	640	160	7/12/99
A/Korea/770/2002†	80	640	1,280	640	640	320	12/2/02
A/WyomingG/03/2003	640	320	1,280	1,280	1,280	320	2/13/03
A/Texas/40/2003	640	1,280	1,280	2,560	1,280	640	10/2/03
A/Oklahoma/8/2004	160	1,280	1,280	2,560	1,280	640	12/8/03
A/Wellington/1/2004	160	320	320	640	320	640	1/26/04
Test antigens							
A/Nepal/1679/04	40	160	1,280	640	2,560	320	7/2/04
A/Nepal/1685/04	40	320	1,280	640	2,560	320	7/2/04
A/Nepal/1670/04	10	160	1,280	320	1,280	320	7/2/04
A/Nepal/1659/04	10	320	640	320	1,280	160	7/2/04
A/Nepal/1660/04	10	160	640	320	2,560	160	7/2/04
A/Nepal/1680/04	20	320	320	320	1,280	160	7/2/04
A/Nepal/1672/04	10	160	320	160	1,280	160	7/2/04
A/Nepal/1678/04	40	160	320	160	1,280	160	7/2/04
A/Nepal/1694/04	10	160	320	160	2,560	160	7/3/04

*Test antigens are considered antigenically different from the reference strain if HI titers show a 4-fold difference. †A/Korea/770/2002 is antigenically equivalent to the A/Fujian/411/02 vaccine strain.

### Molecular Analysis

RT-PCR-based molecular subtyping showed that all 42 specimens were the H3N2 influenza subtype. Twenty-six of the 42 influenza A–positive samples were randomly selected for molecular characterization using direct nucleotide sequencing of the HA gene. The 26 Nepal isolates exhibited 99.8% nucleotide sequence identity and contained the Fujian-like amino acid substitutions at positions 155 (H155T) and 156 (Q156H) in the HA protein (Table 2). Alignment of the 329–amino acid HA protein from 26 isolates obtained from this outbreak with the 2004/05 A/Wyoming/3/03 vaccine strain and previous H3N2 vaccine strains indicated 4 evident amino acid changes present in most of the isolates (Table 2). All 4 amino acid changes observed within most of these outbreak isolates are present within A/California/7/04, a variant strain selected as the H3N2 vaccine strain for the 2005–2006 influenza season.

**Table 2:** Unique hemagglutinin amino acid substitutions from influenza virus isolates obtained during July 2004 influenza outbreak in southeast Nepal compared with 5 vaccine strains*

Virus strain	Amino acid position					
	145 Glycosylation site adjacent to antibody site A	155 Fujian-like lineage amino acid substitution	156 Fujian-like lineage amino acid substitution	189 Antibody site B	226 Antibody site D	227 Antibody site D
A/Nepal Consensus/04†	N	T	H	N	I	P
A/Fujian/411/02	K	T	H	S	V	S
A/Wyoming/3/03	K	T	H	S	I	S
A/Wellington/1/04	K	T	H	N	V	P
A/California/7/04	N	T	H	N	I	P
A/Panama/2007/99	K	H	Q	S	V	S

*N, asparagine; T, threonine; H, histidine; I, isoleucine; P, proline; K, lysine; S, serine; V, valine; Q, glutamine. †Consensus sequence derived from a multiple sequence protein alignment of 26 HA1 hemagglutinin sequences from Nepal.

Of the 26 Nepal strains examined, 24 exhibited a novel lysine-to-asparagine substitution at position 145 in the HA protein (K145N). This substitution is noteworthy because most strains characterized in 2003–2004, including the Fujian/411/2002 vaccine strain, contained a lysine (K) at this position. Prior to this outbreak, the US Department of Defense had only observed K145N substitutions in 6 strains obtained from Ramstein, Germany, (data not shown) in June 2004. Additionally, all 26 Nepal sequences exhibited a serine-to-asparagine substitution at position 189 (S189N) that had also been observed in the 6 isolates from Germany, as well as in a few isolates from Asia characterized at the end of the 2003–2004 influenza season.

Two other substitution mutations in the HA1 hemagglutinin, i.e., valine to isoleucine at position 226 (V226I) and serine to proline at position 227 (S227P), were also observed in 24 (92%) and 26 of 26 of the Nepal isolates, respectively. Both substitutions differ from most influenza A H3N2 field isolates collected in 2003–2004, including the Fujian and Wyoming vaccine strain for 2004–2005 (Table 2).

**Figure 2:**
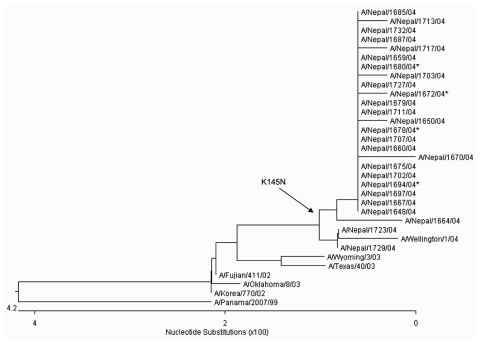
Unrooted phylogenetic analysis of HA1 hemagglutinin nucleotide sequences from 26 Nepal isolates and H3N2 vaccine and reference strains. The Nepal isolates have drifted from the 2004–2005 A/Fujian/411/03 vaccine strain (and A/Wyoming/03/03 vaccine seed strain) and are genetically equivalent to A/California/7/04, the 2005–2006 Northern Hemisphere vaccine strain. A K145N substitution (branch point indicated by the arrow) was observed in 24 of 26 Nepal isolates and represents a genetic marker for the dominant lineage of H3N2 viruses during the 2004–2005 season. Nucleotide and amino acid sequences for all Nepal isolates are available from GenBank under accession no. AY945263–AY945288. The asterisk indicates isolates from Table 2 that were antigenically distinct from A/Wyoming/303.

The phylogeny of H3N2 HA proteins indicates a drifting of the Nepal isolates from the A/Fujian/411/03 and A/Wyoming/03/03 vaccine strains and shows that these outbreak isolates have a higher genetic homology to A/Wellington/1/04, a prototype strain selected as the 2005–2006 Southern Hemisphere H3 vaccine strain (Figure 2). The A/Wellington/1/04 strain contains 2 of the 4 amino acid changes (S227P and S189N) observed in the Nepal isolates, but does not contain the K145N and V226I substitutions.

**Figure 3:**
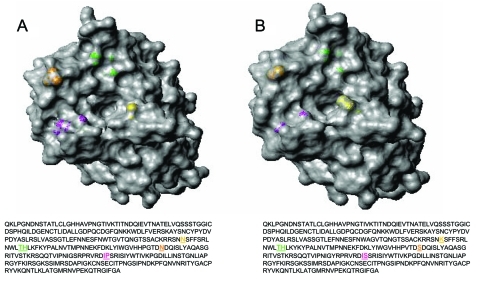
Three-dimensional top view of the HA1 hemagglutinin structures for A) a representative A/Nepal/1648/04 virus and B) vaccine strain A/Wyoming/3/03. Most (24/26) of the Nepal isolates contain a lysine to asparagine substitution (shown in yellow) at position 145 (K145N). Magenta, residues 226 and 227; orange, residue 189; green, residues 155 and 156; yellow, residue 145. Hemagglutinin molecules were generated by using the respective amino acid sequences with MOLMOL (12). A/Nepal/1648/04 is available from GenBank under accession no. AY945264.

Three-dimensional views of influenza HA proteins highlighting amino acid changes in a representative Nepal isolate and the A/Wyoming/3/03 vaccine strains are shown in Figure 3A and B, respectively. The mutation at position 145 (shown in yellow), which is located adjacent to antibody-binding site A and within a known glycosylation site, introduces an asparagine-for-lysine substitution. This substitution results in a more accessible receptor-binding cleft located directly above residue 145 (comparing panels A and B). Located above the receptor-binding pocket is a serine-to-asparagine change (shown in green) that possibly alters the regional surface topography at position 189 within antibody-binding site B. A serine-to-proline mutation at position 227 (shown in magenta) appears to marginally affect the HA surface features. This substitution resides within antibody-binding site D, which corresponds to residues 225–228, which make up the left side of the receptor-binding pocket (14). Interestingly, this proline residue is located within a β barrel (a protein motif consisting of an antiparallel β sheet domain) and does not appreciably alter the predicted protein structure, as shown by the absence of any substantial changes in the computer-modeled, 3-dimensional structure compared with the HA1 of A/Wyoming/3/2003.

## Discussion

The 4 substitutions described represent a growing lineage of influenza A (H3N2) viruses characterized since July 2004. Three amino acid changes are confined within known antibody-binding sites, i.e., the S189N change within antibody-binding site B (4,5) and the V226I and S227P changes residing in antibody-binding site D (4,5). Because of rotational restrictions, a proline substitution at position 227 (S227P) would typically give rise to considerable conformation change; however, this particular substitution is located within a β barrel motif and therefore has little effect on regional protein conformation. Cumulatively, field isolates characterized subsequent to this outbreak continue to exhibit these 4 changes, and they appear to constitute a distinct branch in the phylogeny of HA sequences when compared with H3N2 isolates from the 2003–2004 season.

The K145N mutation represents a change from a charged to uncharged amino acid R group. This change may affect protein-protein interactions since it is immediately adjacent to antibody-binding site A, where neutralizing antibodies have been shown to bind (4,5). Furthermore, since the K145N substitution is located within a glycosylation site, the charge alteration may affect glycosyl transferase activity, which results in altered glycosylation. Differences in glycosylation have been shown to contribute to antigenic variation by preventing antibody binding to antigenic sites (15). Additionally, 3-dimensional analysis suggests this amino acid substitution may also promote enhanced receptor binding since the asparagine R group is shorter, which may make binding requirements less stringent and the receptor cleft more accessible. The 3-dimensional depiction provides a unique regional residue perspective, demonstrating how the rapidly evolving HA surface antigens in the vaccine strain differ at the molecular level. These changes are consistent with both antigenic and genetic data.

Collectively, the clinical isolates obtained from this outbreak in Nepal cannot be considered antigenically distinct from the A/Wyoming/3/03 vaccine strain because only 4 of 9 isolates evaluated exhibited 4-fold lower titers by HI (Table 1). Furthermore, the varying reactivity noted in several isolates from this outbreak having identical HA1 sequences is suggestive that other viral antigens aside from the HA1 protein may have contributed to the antigenic variability observed in the HI panel.

With the exception of A/Nepal/1670/2004 and A/Nepal/1672/2004, all isolates evaluated by HI (Table 1) exhibited identical HA1 amino acid sequences and varying antigenicity profiles to A/Wyoming/03/2003 reference antisera. One explanation for this observation is that genetic differences in other influenza surface proteins contribute to the observed immunoreactivity. Alternative viral surface protein candidates include the neuraminidase, HA2, and M2 glycoproteins, which have been shown to exhibit antigenic properties (16–19).

In this report, we describe the genetic analysis of the HA proteins from viruses obtained from an early season outbreak and compare them to current vaccine strains. Three amino acids changes (S189N, I226V, and S227P) were noted in known (4,5) antibody-binding sites (Table 2). The fourth change (K145N), which was located within a glycosylation site, may enhance viral binding since the smaller asparagine R group is located close to the HA receptor-binding cleft (Figure 3). Phylogenetic analyses show that the Nepal isolates make up a distinct branch in the evolution of H3N2 viruses when they are compared with vaccine and reference strains (Figure 2). However, antigenic data appear more ambiguous, suggesting a multigenic effect that cannot solely be attributed to properties of the influenza HA (Table 1). Studies are in progress to characterize the neuraminidase, M2, and HA2 proteins to determine the molecular basis responsible for antigenicity differences observed within isolates from this outbreak.

The K145N substitution change has become a marker for an increasingly large subset of the Fujian-like viruses. CDC and the US Department of Defense have recently characterized viruses with the K145N change in Singapore, Taiwan, China, Australia, Canada, and the United States. In February 2005, WHO reported the emergence of a new influenza H3N2 strain in the United States. The A/California/7/2004 strain, which was first identified in the United States in September 2004, contains all 4 changes observed in isolates from this Nepalese outbreak. The A/California/7/2004 strain differs by only 1 amino acid in HA1 (which is of no immunologic importance) from most isolates from the outbreak in Nepal. All viruses characterized (≈150 globally isolated strains) subsequent to the preparation of this report (March 2005) by the US Department of Defense are genetically similar in amino acid sequence to these Nepalese strains (and the A/California strain). Most of the isolates (80%) analyzed by CDC since October 2004 are antigenically related to A/California (20), which indicates that this strain has emerged as the dominant influenza A H3N2 strain. These data indicate that these viruses may persist as the dominant strain at the onset of the 2005–2006 influenza season. In February 2005, WHO recommended inclusion of an A/California/7/2004-like strain in the 2005–2006 trivalent influenza vaccine to afford immunologic protection from this variant H3N2 virus. Our findings emphasize the importance of continued molecular surveillance for characterizing emerging influenza drift variants.
